# Should We Consider Them as a Threat? Antimicrobial Resistance, Virulence Potential and Genetic Diversity of *Campylobacter* spp. Isolated from Varsovian Dogs

**DOI:** 10.3390/antibiotics11070964

**Published:** 2022-07-18

**Authors:** Małgorzata Murawska, Monika Sypecka, Justyna Bartosik, Ewelina Kwiecień, Magdalena Rzewuska, Agnieszka Sałamaszyńska-Guz

**Affiliations:** 1 Division of Microbiology, Department of Preclinical Sciences, Institute of Veterinary Medicine, Warsaw University of Life Sciences, Ciszewskiego 8, 02-786 Warsaw, Poland; ewelina_kwiecien1@sggw.edu.pl (E.K.); magdalena_rzewuska@sggw.edu.pl (M.R.); 2 Translational Platform for Regenerative Medicine, Mossakowski Medical Research Institute, Polish Academy of Sciences, Pawińskiego 5, 02-106 Warsaw, Poland; msypecka@imdik.pan.pl; 3 Division of Parasitology and Invasive Diseases, Department of Preclinical Sciences, Institute of Veterinary Medicine, Warsaw University of Life Sciences, Ciszewskiego 8, 02-786 Warsaw, Poland; justyna_bartosik@sggw.edu.pl

**Keywords:** *Campylobacter jejuni*, antimicrobial resistance, virulence factors, PFGE, dogs

## Abstract

Campylobacteriosis seems to be a growing problem worldwide. Apart from the most common sources of numerous *Campylobacter* species, such as poultry and other farm animals, dogs may be an underrated reservoir of this pathogen. Our goal was to establish the frequency of occurrence, antimicrobial resistance, and detection of chosen virulence factor genes in genomes of canine *Campylobacter* isolates. *Campylobacter* isolates frequency in dogs from shelters, and private origin was 13%. All of the tested virulence factor genes were found in 28 of 31 isolates. We determined high resistance levels to the ciprofloxacin and ampicillin and moderate tetracycline resistance. For *C. jejuni* shelter isolates, genetic diversity was also determined using PFGE. Our results indicate that dogs may be the reservoir of potentially diverse, potentially virulent, and antimicrobial-resistant *Campylobacter* strains.

## 1. Introduction

*Campylobacter* spp. is the most common etiological agent of human *gastroenteritis* in Europe, with a reported number of cases of 220,682 in 2019 and a notification rate of 59.7 cases per 100,000 population [1]. Poland has one of the lowest notification rates in the EU (less than 5.8 cases/100,000 population). However, the number of cases of campylobacteriosis in Poland has been growing slowly in recent years, from 552 cases and a 1.4% notification rate in 2013 up to 874 cases and a notification rate of 2.3% in 2017 [1]. Clinical manifestation of campylobacteriosis is non-specific and includes diarrhoea (bloody or watery), abdominal pain and fever. Campylobacteriosis is most frequently a self-limiting disease; however, sometimes antimicrobial treatment is required, especially in immunodeficient patients or when the disease seems to be severe or persistent. Campylobacteriosis may also be followed by complications: gastrointestinal such as Inflammatory Bowel Disease, Barret’s Oesophagus, and extra-gastrointestinal—such as Guillain–Barré Syndrome and Miller–Fisher Syndrome [2]. 

The main sources of numerous *Campylobacter* species are poultry and other farm animals and their products (e.g., meat and milk), contaminated soil and water, as well as wild birds (e.g., gulls) [1,3]. Many studies indicate companion animals (dogs and cats) as a huge, underestimated reservoir of genetically diverse, potentially virulent and antimicrobial-resistant *Campylobacter* species [4,5,6,7]. It has been shown that owning more than one dog or cat, including at least one puppy or kitten, significantly increases the risk of *Campylobacter* infection occurrence in humans [8]. Dogs and cats are mainly asymptomatic carriers of *Campylobacter* spp. Some studies reported that animals with symptoms of gastrointestinal disease (e.g., diarrhoea) as well as animals invaded with intestinal parasites are more likely to be *Campylobacter*-positive. Moreover, increased species richness in diarrheic dogs was highlighted [5,9]. Some factors such as age, stress, living conditions, season, geographic area, model of feeding and breed are indicated as risk factors for *Campylobacter* shedding. Few studies showed that young and senior animals, as well as kennelled or shelter animals are more likely to be *Campylobacter*-positive as well as pure-breed pets in comparison with cross-breed animals [4,5,7,10,11,12,13]. The prevalence of *Campylobacter* spp. in companion animals varies widely from 5.9% to 97% and depends on many factors: animal host species (dog or cat), season of sampling, geographic area, as well as pathogen detection and identification method (culture-based or molecular), study design and the number of samples analysed [4,6,7,8,9,10,11,12,13,14,15,16,17,18,19,20,21,22].

The study goals were to establish the frequency of occurrence, virulence potential and antimicrobial resistance of *Campylobacter* strains isolated from dogs of private and shelter origin. Additionally, the genetic diversity among *Campylobacter jejuni* isolates from shelter dogs was determined.

## 2. Results

### 2.1. Occurrence of Campylobacter spp.

A total number of 196 faecal samples were examined by direct inoculation on mCCD agar, including 124 samples from dogs living in the animal shelter and 72 samples taken from private dogs. 

In total, faecal samples from 26 dogs were *Campylobacter*—positive (13.3%, CI 95%: 8.85–18.83%). The frequency of *Campylobacter* occurrence in shelters and in private dogs was 13.7% (CI 95%: 8.19–21.04%) and 12.5% (CI 95%: 5.88–22.41%), respectively, and did not vary significantly (*p* = 0.81). Eleven of the *Campylobacter*-positive animals (42%) were puppies (≤1 y/o), whereas fifteen (58%) were adults (>1 y/o). Additionally, 16 of the *Campylobacter*-positive animals were concurrently invaded with intestinal parasites: *Giardia intestinalis*, *Toxocara canis*, *Isospora* spp. and non-specified coccidia and nematodes. Exactly 75% of the invaded animals (12 of 16) were from the animal shelter. 

Based on the various colony morphology, two different *Campylobacter* strains were found in the samples from five dogs, including three dogs from a shelter (*C. jejuni*, *C. lari*) and two private dogs (*C. jejuni*); thus, finally, 31 *Campylobacter* spp. strains were tested. PCR and biochemical identification allowed us to identify 19 strains of *Campylobacter jejuni* (9/19 of a shelter origin), nine strains of *Campylobacter lari* (8/9 of a shelter origin) and three strains of *Campylobacter upsaliensis* (3/3 of a shelter origin). A species profile noted for shelter and private dogs varied significantly (*p* = 0.017). Almost 65% of *Campylobacter* spp. strains (*n* = 20) were isolated from shelter dogs, whereas the remaining strains (*n* = 11) were derived from private animals. Neither *Campylobacter helveticus* nor *Campylobacter coli* were isolated. Further information on *Campylobacter*-positive dogs is available in the supplementary material (Table S1). 

### 2.2. Detection of Virulence Factor Genes

PCR screening for selected virulence factor genes confirmed that all tested genes responsible for the cytolethal distending toxin production (*cdtA, cdtB* and *cdtC*) and the motility (*flaA, flaB*) were present in all studied *Campylobacter* spp. strains. The presence of four selected genes responsible for adhesion and internalisation to the host’s cells (*ciaB*, *cadF, pldA* and *flpA*) was confirmed in 28 strains (90%); however, the *cadF* gene was not found in three *C. upsaliensis* strains. The summary of the information on the presence of the virulence factor genes is available in the supplementary material (Table S2).

### 2.3. Phenotypes and Genotypes of the Antimicrobial Resistance 

The Minimum Inhibitory Concentration (MIC) values of the tested antimicrobials were determined using a gradient strip method to establish an antimicrobial resistance phenotype. For selected antimicrobial agents, resistance mechanisms were also studied by PCR detection of appropriate resistance genes. 

**Figure 1:**
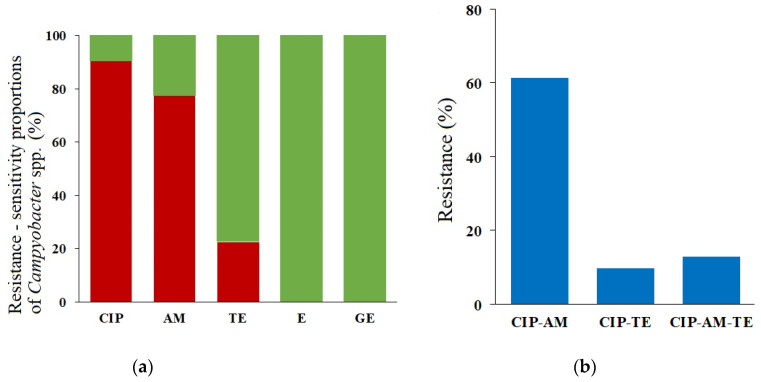
Summary of antimicrobial resistance testing: (**a**) Diagram showing level of susceptibility and resistance to antimicrobial agents, green—susceptible; red—resistant; CIP—ciprofloxacin; TE—tetracycline; AM—ampicillin; E—erythromycin; GE—gentamicin; (**b**) Percentage of resistance to two or more classes of antimicrobials.

The significantly high level of the ciprofloxacin resistance (90.3%; in 28 out of 31 isolates) was determined (Figure 1a). A point mutation C257T (codon 86) in the *gyrA* gene, resulting in amino acid substitution (Thr-86-Ile), was found in the 16 ciprofloxacin-resistant *C. jejuni* strains and two *C. upsaliensis* strains. However, in all resistant *C. lari* and one *C. upsaliensis* isolate, no amino acid substitution was found. 

Seven *C. jejuni* strains (22.6%) were resistant to tetracycline (Figure 1a), and all of them harboured the *tetO* gene in plasmid DNA. 

The resistance to ampicillin was at a high level (77.4%), and it was detected in all *Campylobacter* species, but its determinants in the studied strains were not determined.

No resistance to gentamicin and erythromycin was noted (Figure 1a). 

Twenty-six of thirty-one *Campylobacter* spp. strains (83.9%) were resistant to two or more tested antimicrobial agents, including 4/31 strains (12.9%) classified as multidrug-resistant (resistant to three or more of the antimicrobial classes; MDR) (Figure 1b). Two MDR strains were isolated from dogs of shelter origin and the other two from dogs of private owners. Six resistance patterns could be distinguished, the predominant was CIP^R^ AM^R^ TE^S^, and the less frequent pattern was CIP^S^ AM^R^ TE^S^, found only in one strain (Table 1).

### 2.4. Genetic Diversity of Campylobacter jejuni

Pulsed-field gel electrophoresis (PFGE) was performed to establish the genetic diversity of *C. jejuni* isolates obtained from the shelter dogs. 

**Figure 2:**
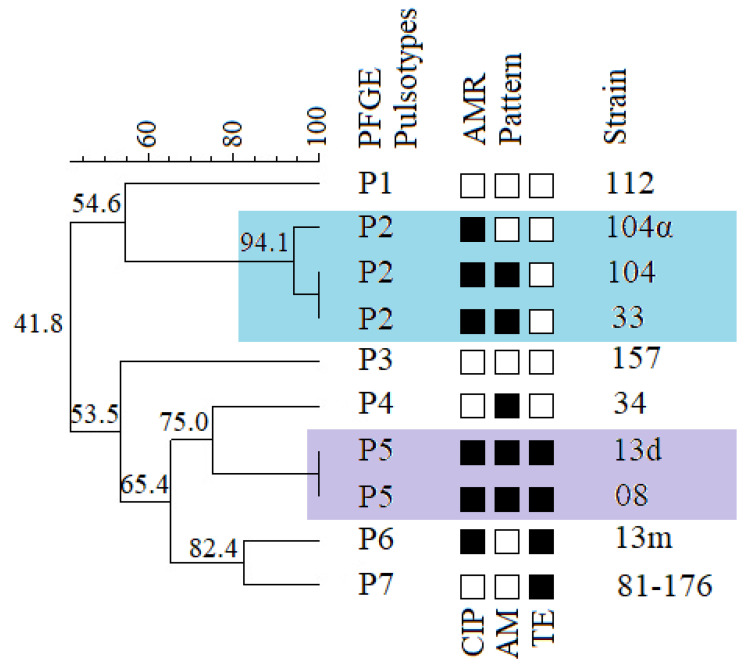
PFGE/SmaI typing of *C. jejuni* strains of shelter origin in compilation with antimicrobial resistance patterns (black square—R; white square—S). A dendrogram was generated using the UPGMA method, a Dice coefficient with a 1.5% tolerance window and a cut-off value of 90%. Bootstrap values are given as a percentage.

Nine *C. jejuni* strains isolated from faeces of the shelter dogs were classified into six pulsotypes (P1–P6). The similarity between those pulsotypes varied from 53.5% to 75% (Figure 2). The Simpson’s index of discrimination of the studied molecular typing method was 0.944 (95% CI: 0.870–1.018).

Interestingly, two *C. jejuni* strains (13d and 13m), isolated from the same dog of shelter origin, have been classified into separate pulsotypes (Figure 2). However, strain 13d clustered together with strain 08 into the pulsotype P5. The pulsotype P2 consisted of three strains; two of them, 104 and 104α, isolated from the same dog were similar at 94.1%, and curiously enough, the third strain isolated from another dog was genetically identical to the strain 104α (Figure 2).

The results of PFGE typing were compared with the antimicrobial resistance patterns. The strains 13d and 13m obtained from the same dog had different resistance patterns. However, strain 104 had a resistance pattern similar to strain 33 and different from the 104α strain, which was isolated from the same dog (Figure 2).

## 3. Discussion

Our study showed that dogs might be an important reservoir of *Campylobacter* species potentially dangerous for human health. The previous studies reported the prevalence of these bacteria in pets’ faeces strongly varied, as well as various virulence potential, antimicrobial resistance, and genetic diversity of *Campylobacter* isolates. It seems that the obtained results may depend on some factors, such as the number of samples, the age and origin of tested animals or the detection and identification methods used.

In this research, we obtained the frequency of the *Campylobacter* spp. occurrence in dog faeces much lower (13.3%) than in most literature reports. In many studies, the prevalence of these bacteria above 20% in dogs was noted [4,7,10,11,12,15,17,18,19]. In a few reports, the prevalence of *Campylobacter* spp. in animals living in shelters varied from 45.4% to 87% [10,11,15]. However, there are also few reports showing similar or lower *Campylobacter* prevalence than in our study, and it varied from 5.9% to 18.3% [6,13,16,21,22,23]. It should be highlighted that in this study, the frequency of *Campylobacter* spp. occurrence did not vary significantly between shelter animals and animals of private owners (13.7% vs. 12.5%). 

Most of the previous studies were focused on two species, *C. jejuni* and *C. coli*, which are the most common causes of human gastroenteritis, but *C. coli* was rarely isolated from dogs [7,8,17,22]. However, Chaban et al. [9] showed, using a quantitive PCR, that dogs may be a reservoir of numerous *Campylobacter* species other than *C. jejuni* and *C. coli*, such as *C. upsaliensis*, *C. hyointestinalis*, *C. showae*, *C. sputorum, C. fetus* and *C. lari*. 

In our study, three species of *Campylobacter*: *C. jejuni*, *C. upsaliensis* and *C. lari* were found. *C. jejuni* was the predominant species, which is in accordance with the literature data [6,7,12,16,17,19,20,22]. Curiously, in this study, the *C. upsaliensis* strains represented only 9.7% of the isolates, even though this species is reported as commonly occurring in *Campylobacter* spp. in dogs, similarly to *C. jejuni* [4,8,12,18,19,20,24]. Interestingly, we identified nine strains of *C. lari*, which was 29% of all isolates. *C. lari* is rarely reported in dogs and cats [7,8,19,20] but is mostly associated with animals living in coastal regions and the marine environment (gulls, molluscs, aquatic mammals, and birds) [3,25]. Thus, such a high frequency of this species in dogs in the central region of Poland (Warsaw and the surrounding area) is difficult to explain. However, we lack information on the history of the origin of those dogs. Eight of nine *C. lari* strains were isolated from shelter dogs, as well as all *C. upsaliensis* strains. It suggests that animals kept in shelters potentially may be a reservoir of more diverse *Campylobacter* species than dogs or cats of private owners. We did not isolate any *C. coli* and *C. helveticus* strains, which is consistent with some previous reports [4,11,13,16,19]. 

The motility, cytolethal distending toxin production and ability to adhere and invade the host’s cells are pointed to as the main virulence factors among *Campylobacter* species. Most research focused on detection of genes encoding flagellar proteins (FlaA, FlaB, FlaC), subunits of cytolethal distending toxin (CdtA, CdtB, CdtC), adhesins (CadF, FlpA) and invasion factors (CiaB, IamA). Some of them also indicated a product of the *pldA* gene encoding outer membrane phospholipase, which is involved in the invasion and colonisation process [24,26,27,28,29,30,31]. 

In all examined strains in this study, both the presence of the *flaA* and *flaB* genes was detected. They are encoding two proteins, FlaA and FlaB, responsible for the flagellum’s filament formation and thus are responsible for the *Campylobacter* motility. These results are consistent with the available literature data, where a high prevalence of the *flaA* gene, between 82–100% in human, canine and feline isolates, was detected [6,24,26,28]. 

The other important virulence factor of *Campylobacter* spp. is the cytolethal distending toxin (CDT) with DNase activity, which is composed of three subunits encoded by the *cdtA, cdtB* and *cdtC* genes. While the CdtB subunit is an active compound (DNase I), CdtA and CdtC mediate in binding and internalisation to the host’s cells [31]. Therefore, most of the studies focused on the presence of the *cdtB* gene [24,26,29,32], but some of them investigated all of the three genes or the *cdtABC* cluster [28,30]. Unlike the *flaA* gene, the prevalence of CDT encoding genes varied from 29.5–100% [6,24,28]. In our study, *cdtA, cdtB* and *cdtC* were found in all of the examined strains belonging to *C. jejuni*, *C. lari* and *C. upsaliensis* species; thus, a fully functional toxin may be produced by those isolates. 

The ability to adhere to and invade of host’s enterocytes is also crucial for the pathogenesis of campylobacteriosis. The *Campylobacter* adhesion proteins, CadF and FlpA (fibrin-like peptide A), are outer membrane proteins responsible for adhesion initiation by binding to fibronectin of epithelium [27,31]. Products of the *pldA* gene, phospholipase A, and CiaB protein are involved in the internalisation of *Campylobacter* into the host’s cells [27,31]. The frequency of *flpA*, *ciaB* and *pldA* genes obtained in our study was 100%, while the *cadF* gene occurred in 90.3% of the isolates overall. We did not find *cadF* in *C. upsaliensis* strains, but 100% of the *C. jejuni* and *C. lari* strains were positive for this gene. The gene *cadF* was usually detected in 100% of isolates from different sources: dogs, cats, broilers, pigs, cattle, and humans [6,24,26,30,32]. Only in research by Selwet *cadF* was found in 83.6% of isolates from dogs [28]. 

Almost the 100% frequency of selected genes occurrence, responsible for adhesion and invasion process makes obtained isolates potentially able to colonise and damage intestinal epithelium, which may result in symptomatic infection development. 

As for all emerging pathogens, the antimicrobial resistance (AMR) in *Campylobacter* spp. has been a growing problem over the years. For the *Campylobacter* genus, increasing resistance to fluoroquinolones, quinolones, tetracyclines and penicillins is constantly observed. Preventive administration of those antimicrobials in animal husbandries (chicken and turkey broilers, pigs and cattle) and pet stores (puppies and kittens) may be indicated as one of the main reasons for increasing AMR and more frequent occurrence of multidrug-resistant strains [33,34]. Thus, it seems reasonable to monitor AMR and the occurrence of multidrug resistance (MDR) in *Campylobacter* strains of pet origin.

In this study, antimicrobial susceptibility testing was performed for all isolated *Campylobacter* spp. strains. It is worth noting that in the case of *Campylobacter* antimicrobial susceptibility testing, the interpretation of the results may be problematic due to the fact that breakpoints are available for few antimicrobials and are only established for *C. jejuni* and *C. coli*, as these species are the main etiological factors of human campylobacteriosis.

A very high level of ciprofloxacin resistance (90.3%) was noted among the studied *Campylobacter* spp. strains (Figure 1). Our results are similar to those performed on canine and feline isolates in Iran, where the ciprofloxacin resistance was determined at the level of 75% [13]. The main mechanism of resistance to fluoroquinolones is the weakening of the antimicrobial binding to the so-called quinolone-binding pocket at the GyrA subunit of DNA gyrase, caused by amino acid substitution [35,36,37]. We found the most common point mutation, C257T, in codon 86 of the *gyrA* gene, resulting in amino acid substitution (Thr-86-Ile) in 19 (67.9%) of the ciprofloxacin-resistant strains, 17 *C. jejuni* and two *C. upsaliensis* strains. No other mutations in the *gyrA* gene have been found in the studied ciprofloxacin-resistant strains.

In this study, the level of tetracycline resistance was at 22.6%, which may be considered moderate in comparison with varied data from a few reports on tetracycline resistance in canine and feline *Campylobacter* strains. In some studies, a low tetracycline resistance level (0% to 8.6%) was noted, however other studies revealed high levels of tetracycline resistance, up to 87.5% [13,14,22,28]. 

Tetracycline activity is based on binding to the ribosome and inhibiting the protein synthesis via blocking the accommodation of aminoacyl-tRNA into the A site of the ribosome. One of the main mechanisms of tetracycline resistance is associated with the presence of ribosomal protection proteins (RPP’s) encoded by *tet* genes [38]. Among the *Campylobacter* genus, the presence of the *tetO* gene on a plasmid or chromosomal DNA, encoding Tet(O) RPP, is the most widespread mechanism of tetracycline resistance [39]. In this study, in all seven tetracycline-resistant *Campylobacter* strains, the *tetO* gene was found in plasmid DNA. This result is in accordance with the literature data [30,40,41,42], where Tet(O) was found as a main tetracycline resistance determinant. 

β-lactam antibiotics are not commonly used for campylobacteriosis treatment, whereas macrolides (azithromycin in particular) are now applied as a gold standard [30]. However, few studies included the determination of the β-lactam resistance; thus, we decided to perform susceptibility testing for ampicillin. A total of 77.4% of the studied strains were ampicillin-resistant, which is much more than found in literature data, where the prevalence of aminopenicillin resistance ranged from 25% to 58.1% [13,22,30,43]. However, the study of Rozynek et al. showed that the level of ampicillin resistance increased over the years from 8% to 35.5% and from 5.8% to 30.4% in human and broiler isolates, respectively [40]. 

We did not obtain any macrolide and aminoglycoside resistant strains of *Campylobacter* spp. Most of the studies reported low levels of resistance to those two classes of antimicrobials, and, in most cases, only single isolates were resistant [23,28,29,30,40,41,43]. 

Despite the fact that we did not determine any resistance to macrolides or aminoglycosides, four of the isolated strains were classified as multidrug-resistant because they revealed the resistance to three classes of antimicrobial agents: fluoroquinolones, tetracyclines and β-lactams. The report of Montgomery et al. on the outbreak of MDR *C. jejuni* in the United States after puppy exposure indicated that preventive usage of antimicrobials in animals in pet stores could contribute to developing multidrug resistance and may lead to more outbreaks of potentially untreatable infections in humans [33]. 

As the animal shelter is a closed environment in which the strains of bacteria can circulate within the hosts, we decided to perform PFGE typing of nine strains of *C. jejuni* isolated from shelter dogs and additionally the reference strain *C. jejuni* 81176 of human origin, to determine genetical diversity among those strains. We showed that one dog might be a source of different *C. jejuni* strains, e.g., genetically diverse strains 13d and 13m from one animal, belonging to the two different pulsotypes. Our results also indicate that strains clustering into one pulsotype share identical or very similar antimicrobial resistance patterns (Figure 2) which comes along with the research of Du et al. [44] on human and poultry isolates but stays in contrast with the results of Bakhshi et al. [45] where the genetically similar isolates of poultry meat origin showed different AMR patterns. It has been confirmed that *Campylobacter* spp. strains could spread between animals in a shelter.

Most of the studies on the genetic diversity of animal *Campylobacter* spp. strains were focused on the investigation of isolates from slaughter animals (chicken broilers, bovine meat, pig fatteners) due to the fact that meat contamination is indicated as the most common source of pathogenic *Campylobacter* for humans. Those studies showed that mentioned animal-origin isolates are genetically highly diverse [46,47,48,49,50]. However, little is still known about the genetic diversity of canine *Campylobacter* strains. Our data and previously published data clearly indicate that dogs and cats may be a reservoir of diverse *Campylobacter* species and an important source of infections caused by these pathogens in humans [6,7]. 

## 4. Materials and Methods

### 4.1. Sampling and Shipment

Samples of faeces were taken from 196 dogs, including 124, mostly healthy, dogs from one of the Varsovian animal shelters and 72 dogs of private owners, subjected to veterinary cabinets in the Warsaw agglomeration (Poland) due to needing to perform faeces examination. The study included animals, both males and females, aged from one month to 16 years old. The samples were collected between February 2019 and June 2020. 

Faecal samples in sterile containers were stored at a temperature of 2–8 °C until shipping and were delivered to the microbiological laboratory within 24–48 h from sampling. Apart from a bacteriological study, additionally, a parasitology examination using the routine methodology was performed for all samples.

### 4.2. Isolation and Identification of Campylobacter spp.

Specimens were streaked on selective mCCD agar plates (GRASO Biotech, Starogard Gdański, Poland) using sterile cotton swabs, followed by two reducing streaks with a laboratory wire loop. Plates were incubated at 42 °C for 48 h under microaerophilic conditions using the GasPak Campy Container System (BD Biosciences, USA). After incubation, one or two colonies (two colonies were taken if they differed) from each selective plate were cultured on Columbia Blood Agar (GRASO Biotech, Starogard Gdański, Poland) at 42 °C for 48 h under microaerophilic conditions. When pure cultures were obtained, preliminary identification to the genus level was performed based on specific colony morphology, cell morphology, and the motility observed by phase-contrast microscopy, as well as catalase and oxidase test results. Preliminary identification was followed by DNA isolation using the Genomic Mini isolation kit (A&A Biotechnology, Gdańsk, Poland), performed according to the manufacturer’s instructions. Further recognition of isolates to the species level was performed using PCR with species-specific primers (Table 2). Reaction conditions were as follows: pre-denaturation at 95 °C for two minutes; 25 cycles of denaturation at 95 °C for 30 s, primer annealing for 30 s, elongation at 72 °C for 30 s or 60 s (for *C. upsaliensis* and *C. helveticus* identification); and an additional elongation step at 72 °C for five minutes; annealing temperatures for all used primers are presented in Table 2. For positive control, *Campylobacter jejuni* 81-176 ATCC^®^ BAA2151 and *Campylobacter coli* 605 strains were used. Identification of *C. lari* and *C. upsaliensis* strains was also confirmed with the API Campy test (bioMérieux, Marcy-l’Étoile, France), following the manufacturer’s protocol. 

### 4.3. Virulence Factor Genes Occurrence

For identified strains of *Campylobacter* spp., PCR assays were performed to detect selected virulence factor genes: *flaA*, *flaB*, *cdtA*, *cdtB*, *cdtC*, *ciaB*, *pldA*, *cadF* and *flpA*. The used primers are listed in Table 3. A template chromosomal DNA was isolated using the Genomic Mini isolation kit (A&A Biotechnology, Gdańsk, Poland). For positive control, *Campylobacter jejuni* 81-176 ATCC^®^ BAA2151 was used. All reactions were performed under the following conditions: pre-denaturation at 95 °C for two minutes; 30 cycles of denaturation at 95 °C for 30 s, primer annealing for 30 s, elongation at 72 °C for 30 s (90 s for the *flaA*); an additional elongation step at 72 °C for five minutes; annealing temperatures for the used primers are presented in Table 3.

### 4.4. Antimicrobial Susceptibility Testing

Antimicrobial susceptibility to ciprofloxacin (CIP), tetracycline (TE), gentamicin (GE), erythromycin (E) and ampicillin (AM) was tested by MIC determination using the Etest gradient strips (bioMérieux, Marcy-l’Étoile, France), according to the CLSI or EUCAST guidelines [59,60]. Briefly, bacterial suspensions were prepared in 1 mL of sterile buffered saline to obtain 0.5 McFarland density and streaked on Columbia Blood Agar plates with a sterile cotton swab. Plates were dried for a few minutes, then gradient strips were placed, and cultures were incubated at 42 °C for 48 h under microaerophilic conditions. After incubation, the MIC values were read. Results of antimicrobial susceptibility testing were interpreted according to the CLSI and EUCAST guidelines [59,60]. *Campylobacter jejuni* 81-176 ATCC^®^ BAA2151 strain was used as the quality control. All tested antimicrobials and interpretation criteria used are listed in Table 4.

### 4.5. Investigation of Antimicrobial Resistance Mechanisms

For tetracycline-resistant strains, PCR assays using both chromosomal and plasmid DNA were performed to detect the *tetO* gene. A plasmid DNA was isolated using a Plasmid Mini isolation kit (A&A Biotechnology, Gdańsk, Poland) and a chromosomal DNA using the Genomic Mini isolation kit (A&A Biotechnology, Gdańsk, Poland), according to manufacturer protocols.

For ciprofloxacin-resistant strains, a 290-bp fragment of the *gyrA* gene (codons 32-127) was amplified by PCR using genomic DNA as a template. The following conditions for both reactions were applied: pre-denaturation at 95 °C for two minutes; 25 cycles of denaturation at 95 °C for 30 s, primer annealing for 30 s, elongation at 72 °C for 30 s (for *tetO* and *gyrA* amplification); an additional elongation step at 72 °C for three minutes; primer sequences and annealing temperatures are listed in Table 3. PCR products of *gyrA* were sequenced (Genomed, Poland) and then analysed for point mutations using a DNA Baser Assembler software v. 5.11.3 (Heracle Biosoft SRL, Mioveni, Romania).

### 4.6. Pulsed-Field Gel Electrophoresis (PFGE)

Pulse-field gel electrophoresis (PFGE) was performed only for *C. jejuni* strains isolated from the shelter dogs, according to Ribot et al. rapid PFGE protocol for subtyping of *Campylobacter jejuni* [61]. Briefly, pure cultures of the strains were streaked on the Columbia Agar plates (GRASO, Starogard Gdański, Poland) and cultured at 42 °C for 24 h in microaerophilic conditions. After incubation, bacterial suspensions were prepared in sterile buffered saline (OD_600_ = 0.6–0.8) with the addition of a proteinase K (A&A Biotechnology, Gdańsk, Poland) and then were mixed with 2% low melting point agarose (Bio-rad, Hercules, CA, USA) in a ratio of 1:1, and agarose plugs were prepared on glass slides. Plugs were incubated in the cell lysis buffer with proteinase K and then washed once with sterile deionised water and three times with Tris-EDTA (TE) buffer. Plugs were stored in TE buffer at 4 °C until digestion. Digestion was preceded by equilibration of plugs in a solution containing restriction enzyme buffer and sterile deionised water. Plugs were then digested with 40 U of *Sma*I enzyme (Thermo Fisher Scientific, Waltham, MA, USA) at 25 °C for two hours, then washed in 0.5× TRIS-borate-EDTA (TBE) buffer once and equilibrated in 200 µL of fresh 0.5× TBE buffer. Electrophoresis was performed in 1.5% agarose in 0.5× TBE buffer on the CHEF-DR II apparatus (Bio-Rad, Hercules, CA, USA) under the following conditions: an initial pulse time of 6.8 s, a final pulse time of 38.4 s, gradient 6 V/cm, 19 h, angle 120°. The gel was stained with ethidium bromide (0.5 µg/mL) and washed in distilled water, and then visualised on a Gel Doc™ EZ Imaging System with Image Lab software v. 5.2.1 (Bio-Rad, Hercules, CA, USA). The images were analysed using BioNumerics v. 7.6 software (Applied Maths, Sint-Martens-Latem, Belgium). Obtained patterns were compared, and a UPGMA dendrogram was generated, using a Dice coefficient with a 1.5% tolerance window and 90% cut-off value. Simpson’s index of discrimination was also calculated. The analysed strains were compared to the reference *Campylobacter jejuni* 81-176 (ATCC^®^ BAA2151) strain.

### 4.7. Statistical Analysis

Statistical analysis was performed using a IBM SPSS software Statistics for Windows, version 28 (IBM Corp., Armonk, N.Y., USA) software [62]. Confidence intervals were calculated using a Sample Size Calculators online tool [63].

## 5. Conclusions

Companion animals may be a potential source of *Campylobacter* strains causing zoonotic infections in humans; therefore, it seems reasonable to monitor the occurrence of these bacteria in pets. In this study, we obtained the relatively low frequency of the *Campylobacter* spp. occurrence in dogs; however, the isolated strains seem to be potentially virulent and antimicrobial-resistant. 

Since, from the epidemiological point of view, *C. jejuni* and *C. coli* are a significant threat to human health, most studies are focused only on those species and are based on phenotypic bacteriological methods, while it was shown that molecular detection and identification methods are more effective and may allow detection of a higher prevalence and a species richness. Results of this research showed that dogs might play a role as reservoirs of different, potentially virulent *Campylobacter* species, which can be antimicrobial-resistant and even multidrug-resistant. Therefore, it seems reasonable to monitor the dissemination of various *Campylobacter* species in different populations of dogs, including shelters and veterinary cabinets from a large area, to obtain more meaningful results, and even though *C. lari* has been connected with single cases of human campylobacteriosis, it seems important to control its occurrence, as many virulence factors and antimicrobial resistance mechanisms are conserved and similar to those present in *C. jejuni*.

## Figures and Tables

**Table 1:** Frequency of occurrence of the AMR patterns obtained in this study.

Resistance Profile	Number of Isolates	Frequency [%]	Species Included [%]
CIP^R^ AM^R^ TE^S^	19	61.3	*C. jejuni* (47.4)*, C. lari* (15.8)*, C. upsaliensis* (36.8)
CIP^R^ AM^R^ TE^R^	4	12.9	*C. jejuni* (100)
CIP^R^ AM^S^ TE^R^	3	9.6	*C. jejuni* (100)
CIP^R^ AM^S^ TE^S^	2	6.5	*C. jejuni* (100)
CIP^S^ AM^S^ TE^S^	2	6.5	*C. jejuni* (100)
CIP^S^ AM^R^ TE^S^	1	3.2	*C. jejuni* (100)
Total	31		

**Table 2:** Primers used in this study for *Campylobacter* species identification.

Target Species	Primer	Amplicon Size [bp]	Primer Sequence 5′-3′	Tm * [°C]	Ref.
*C. jejuni*	mapAF	604	ATGTTTAAAAAATTTTTG	55	[51]
	mapAR		AAGTTCAGAGATTAAACTAG		
*C. upsaliensis*	CHCU146F	879	GGGACAACACTTAGAAATGAG	55	[52]
	CU1024R		CACTTCCGTATCTCTACAGA		
*C. helveticus*	CHCU146F	1226	GGGACAACACTTAGAAATGAG	52	[52]
	CH1371R		CCGTGACATGGCTGATTCAC		
*C. lari*	lpxAF	233	TRCCAAATGTTAAAATAGGCGA	50	[53]
	lpxAR		CAATCATGDGCDATATGASAATAHGCCAT		
*C. coli*	Mu3	364	AGGCAAGGGAGCCTTTAATC	61	[51]
	Mu4		TATCCCTATCTACAATTCGC		

* Tm—temperature of primer annealing.

**Table 3:** Primers used in this study for the detection of virulence factor genes and determination of the molecular mechanisms of antimicrobial resistance.

Target Gene	Primer	Amplicon Size [bp]	Primer Sequence 5′- 3′	Tm * [°C]	Ref.
*flaA*	flaA-F	1728	GGATTTCGTATTAACACAAATGGTGC	45	[54]
	flaA-R		CTGTAGTAATCTTAAAACATTTTG		
*flaB*	fB1	260	AAGGATTTAAAATGGGTTTTAGAATAAACACC	54	[55]
	fA2		GCTCATCCATAGCTTTATCTGC		
*cdtA*	cdtA-F	370	CCTTGTGATGCAAGCAATC	46	[56]
	cdtA-R		ACACTCCATTTGCTTTCTG		
*cdtB*	cdtB-F	620	CAGAAAGCAAATGGAGTGTT	47	[57]
	cdtB-R		AGCTAAAAGCGGTGGAGTAT		
*cdtC*	cdtC-F	182	TTGGCATTATAGAAAATACAGTT	46	[57]
	cdtC-R		CGATGAGTTAAAACAAAAAGATA		
*ciaB*	ciaB-F	527	TGCGAGATTTTTCGAGAATG	47	[58]
	ciaB-R		TGCCCGCCTTAGAACTTACA		
*pldA*	pldA-F	385	AAGAGTGAGGCGAAATTCCA	49	[58]
	pldA-R		GCAAGATGGCAGGATTATCA		
*flpA*	flpAF	1017	GCTTTTGAATGGGAGTCTTTATAT	49	This study
	flpAR		ATCAATAGCAATAACTTCATAACTATA		
*cadF*	cadF_F	580	TTTGAGTGCTATTAAAGGTATTG	47	This study
	cadF_R		TCTTTCTGAAAGCTTTTGATTATA		
*cadF* *(C. lari)*	cadF_LF	589	GCGCACGACCTTCTTTAGT	50	This study
	cadF_LR		GCTTATGAAAATAAAAGCGGTATG		
*cadF* *(C. upsaliensis)*	cadF_UF	510	CTCTCTTGGTTCTTCAGGACA	52	This study
	cadF_UR		GATAATCGCTATGCACCAGGGA		
*tetO*	tetO_F	559	GGCGTTTTGTTTA	49	[37]
	tetO_R		ATGGACAACCCGACAGAAGC		
*gyrA*	gyrA_F	290	ATTATAGGTCGTGCTTTGCCT	50	This study
	gyrA_R		GCTTCAGTATAACGCATCGCA		

* Tm—temperature of primer annealing

**Table 4:** Interpretation criteria (breakpoints) for antimicrobial susceptibility determination.

Antimicrobial Agent	Antimicrobial Class	Concentration Range Tested [mg/L]	Breakpoints for MIC Testing		
			S ≤	I	R ≥
CIP ^b^ *	Fluoroquinolones	0.002–32	0.001	-	0.5
TE ^a^	Tetracyclines	0.016–256	4	8	16
AM ^b^	β-lactams	0.016–256	2	-	8
E ^a^	Macrolides	0.016–256	8	16	32
GE ^b^	Aminoglycosides	0.016–256	0.5	-	0.5

* Interpretation according to: ^a^ CLSI or ^b^ EUCAST

## Data Availability

The data presented in this study are available on request from the corresponding authors.

## Supplementary Materials

The following supporting information can be downloaded at: https://www.mdpi.com/article/10.3390/antibiotics11070964/s1, Table S1: The summary of information on *Campylobacter*-positive dogs; Table S2: The summary of information on the presence of the selected virulence factor genes among *Campylobacter* isolates.
