# Supplementary material for: Should We Consider Them as a Threat? Antimicrobial Resistance, Virulence Potential and Genetic Diversity of Campylobacter spp. Isolated from Varsovian Dogs
Source: Antibiotics (Basel). 2022 Jul 18;11(7):964. doi: 10.3390/antibiotics11070964 (PMC9311969; doi:10.3390/antibiotics11070964)
Supplement: Supplementary file 1 [file antibiotics-11-00964-s001.zip › Table S1.pdf]

Table S1. The summary of information on *Campylobacter*-positive dogs.

Dog ID	Origin	Sex	Age	Parasites	*Campylobacter* strain designation	*Campylobacter* species
03	Shelter	M	2 years	+	3	*C. lari*
08	Shelter	M	5 years	+	8	*C. jejuni*
10	Shelter	F	3 years	+	10sz<br>10b	*C. lari*<br>*C. lari*
13	Shelter	F	2 years	+	13d<br>13m	*C. jejuni*<br>*C. jejuni*
24	Private	F	1 year	-	24	*C. lari*
26	Private	M	12 years	-	26b	*C. jejuni*
28	Shelter	M	12 years	-	28	*C. upsaliensis*
30	Shelter	M	5 months	-	30	*C. lari*
31	Shelter	M	5 months	-	31	*C. lari*
33	Shelter	F	2,5 years	+	33	*C. jejuni*
34	Shelter	M	1,5 years	+	34	*C. jejuni*
35	Shelter	M	8 months	-	35	*C. upsaliensis*
37	Shelter	F	1,5 years	+	37	*C. upsaliensis*
83	Shelter	M	1 year	+	83	*C. lari*
85	Private	M	8 months	+	85<br>85sz	*C. jejuni*<br>*C. jejuni*
89	Shelter	F	1 year	+	89	*C. lari*
102	Private	F	10 years	-	102	*C. jejuni*
104	Shelter	M	7 years	+	104<br>104α	*C. jejuni*<br>*C. jejuni*
112	Shelter	M	8 years	+	112	*C. jejuni*
132	Shelter	F	5 years	-	132	*C. lari*
157	Shelter	F	2 months	+	157	*C. jejuni*
179	Private	M	10 months	-	179	*C. jejuni*
183	Private	F	1 year	+	183	*C. jejuni*
209	Private	M	2 years	+	209	*C. jejuni*
267	Private	M	5 months	+	267	*C. jejuni*
292	Private	F	10 years	-	292a<br>292b	*C. jejuni*<br>*C. jejuni*
