# Supplementary material for: Should We Consider Them as a Threat? Antimicrobial Resistance, Virulence Potential and Genetic Diversity of Campylobacter spp. Isolated from Varsovian Dogs
Source: Antibiotics (Basel). 2022 Jul 18;11(7):964. doi: 10.3390/antibiotics11070964 (PMC9311969; doi:10.3390/antibiotics11070964)
Supplement: Supplementary file 1 [file antibiotics-11-00964-s001.zip › Table S2.pdf]

Table S2. The summary of information on the presence of the selected virulence factor genes among *Campylobacter* isolates.

**Virulence factor**	**Gene**	**Occurrence [%]**	***Campylobacter* species**
Motility	*flaA*	100.0	*C. jejuni, C. lari, C. upsaliensis*
	flaB	100.0	*C. jejuni, C. lari, C. upsaliensis*
CDT subunits	*cdtA*	100.0	*C. jejuni, C. lari, C. upsaliensis*
	cdtB	100.0	*C. jejuni, C. lari, C. upsaliensis*
	cdtC	100.0	*C. jejuni, C. lari, C. upsaliensis*
Adhesion & Invasion	*ciaB*	100.0	*C. jejuni, C. lari, C. upsaliensis*
	cadF	90.0	*C. jejuni, C. lari*
	pldA	100.0	*C. jejuni, C. lari, C. upsaliensis*
	flpA	100.0	*C. jejuni, C. lari, C. upsaliensis*
